# Retrosternal gastric reconstruction after esophagectomy using the “waterfall” method for posterior mediastinal dead space filling

**DOI:** 10.1007/s10388-026-01204-4

**Published:** 2026-04-08

**Authors:** Takushi Yasuda, Osamu Shiraishi, Hiroaki Kato, Yoko Hiraki, Naoko Kounami, Masuhiro Terada, Atsushi Yamada, Masashi Kohda, Tomoya Nakanishi, Atsushi Yasuda, Masayuki Shinkai, Yutaka Kimura, Motohiro Imano

**Affiliations:** 1 Department of Surgery, Faculty of Medicine, Kindai University, 1-14-1, Mihara-Dai, Minami-Ku, Sakai, Osaka 590-0197 Japan; 2 Department of Gastroenterological Surgery, Kindai University Nara Hospital, Nara, Japan

**Keywords:** Esophageal reconstruction, Omental flap, Gastric conduit, Anterior mediastinal route, Surgical technique

## Abstract

**Background:**

Advanced esophageal squamous cell carcinoma (ESCC) may require combined resection or result in non-curative resection, increasing the risk of severe complications related to the posterior mediastinal dead space created after esophagectomy, such as airway instability and mediastinitis. Although filling this space with the greater omentum via the posterior mediastinal route is commonly used, this approach may impair gastric conduit alignment and function due to intrathoracic negative pressure. We developed a novel technique to overcome these limitations.

**Methods:**

We retrospectively reviewed 20 patients who underwent esophagectomy with gastric conduit reconstruction and additional omental transposition using a novel “waterfall” method between 2012 and 2022. Omental transposition was selectively added in cases involving combined resection of adjacent organs or non-curative resection, in which the posterior mediastinal dead space was considered to increase postoperative complication risk. In this technique, the greater omentum is elevated to the neck with the gastric conduit via the retrosternal route and then pulled down from the anterior mediastinum to fill the posterior mediastinal dead space. Surgical outcomes, postoperative complications, and conduit-related symptoms were evaluated.

**Results:**

Sixteen patients had cT4b disease and four had cT3 disease. Curative resection was achieved in 12 patients, while 8 underwent non-curative resection. No serious complications such as airway necrosis, airway–mediastinal fistula, or mediastinitis were observed, and no patients developed gastric conduit dysfunction.

**Conclusions:**

The waterfall method is a safe and feasible technique for filling the posterior mediastinal dead space after esophagectomy while preserving gastric conduit alignment and function in high-risk ESCC cases.

**Supplementary Information:**

The online version contains supplementary material available at 10.1007/s10388-026-01204-4.

## Introduction

Esophageal squamous cell carcinoma (ESCC) is a highly malignant disease characterized by aggressive tumor growth. Because the esophagus is centrally located in the mediastinum and anatomically adjacent to vital organs, tumor progression frequently results in invasion of surrounding structures, including the trachea, main bronchus, and aorta. According to the Japanese Esophageal Cancer Registry in 2016, 9.2% of patients were initially diagnosed with cT4b disease [1]. In most such cases, intensive induction therapy—such as chemoradiotherapy and/or triplet chemotherapy—is indicated with curative intent. When sufficient downstaging is achieved and the tumor is considered potentially resectable, conversion surgery may be performed, provided the patient’s general condition allows. However, accurate intraoperative assessment of tumor resectability remains challenging, particularly at an early stage of the operation. As a result, combined resection of infiltrated adjacent organs may ultimately be required in some cases, and it has been reported that 2.8% of esophagectomies result in non-curative (R2) resection [1].

When tumor infiltration does not extend to the tracheal or bronchial smooth muscle, the connective tissue surrounding the airway membranous portion may be resected en bloc with the tumor to secure an adequate surgical margin. In such cases, the resected membranous portion becomes thin and fragile due to loss of supporting structures, resulting in direct exposure to the post-esophagectomy cavity. This loss of structural support predisposes the airway to excessive respiration-dependent deformation and potential collapse. In contrast, in cases resulting in R2 resection, the ulcer bed of residual tumor—contaminated with bacterial flora from the oral cavity and pharynx—remains within the mediastinum and is exposed to postoperative exudate. This environment promotes bacterial proliferation and can lead to severe deep mediastinitis. Once any of these complications develops, they may be fatal and therefore must be avoided whenever possible. Accordingly, it is critically important to fill the posterior mediastinal dead space created after esophagectomy with viable tissue. This is necessary both to reinforce the fragile airway membranous portion after combined resection of peritracheal connective tissue and to prevent the spread of infection by covering residual tumor tissue in cases of R2 resection.

In esophageal reconstruction, the stomach is typically mobilized to the neck to restore alimentary continuity, together with the greater omentum. When posterior mediastinal dead space requires filling, the gastric conduit is usually elevated via the posterior mediastinal route. However, because priority is given to packing the central posterior mediastinal cavity with the greater omentum, the gastric conduit is often displaced dorsomedially into the right thoracic cavity and remains subject to intrathoracic negative pressure [2]. As a result, dilation and/or displacement of the gastric conduit may occur, leading to impaired transit and delayed gastric emptying [3, 4].

To overcome these issues, we developed a novel procedure in which the greater omentum is elevated together with the gastric conduit via the retrosternal route and subsequently pulled down from the anterior mediastinum to fill the posterior mediastinal dead space. In this study, we report the feasibility, safety, and clinical usefulness of this technique, which we have termed the “waterfall” method.

## Methods

### Patients

**Fig. 1:**
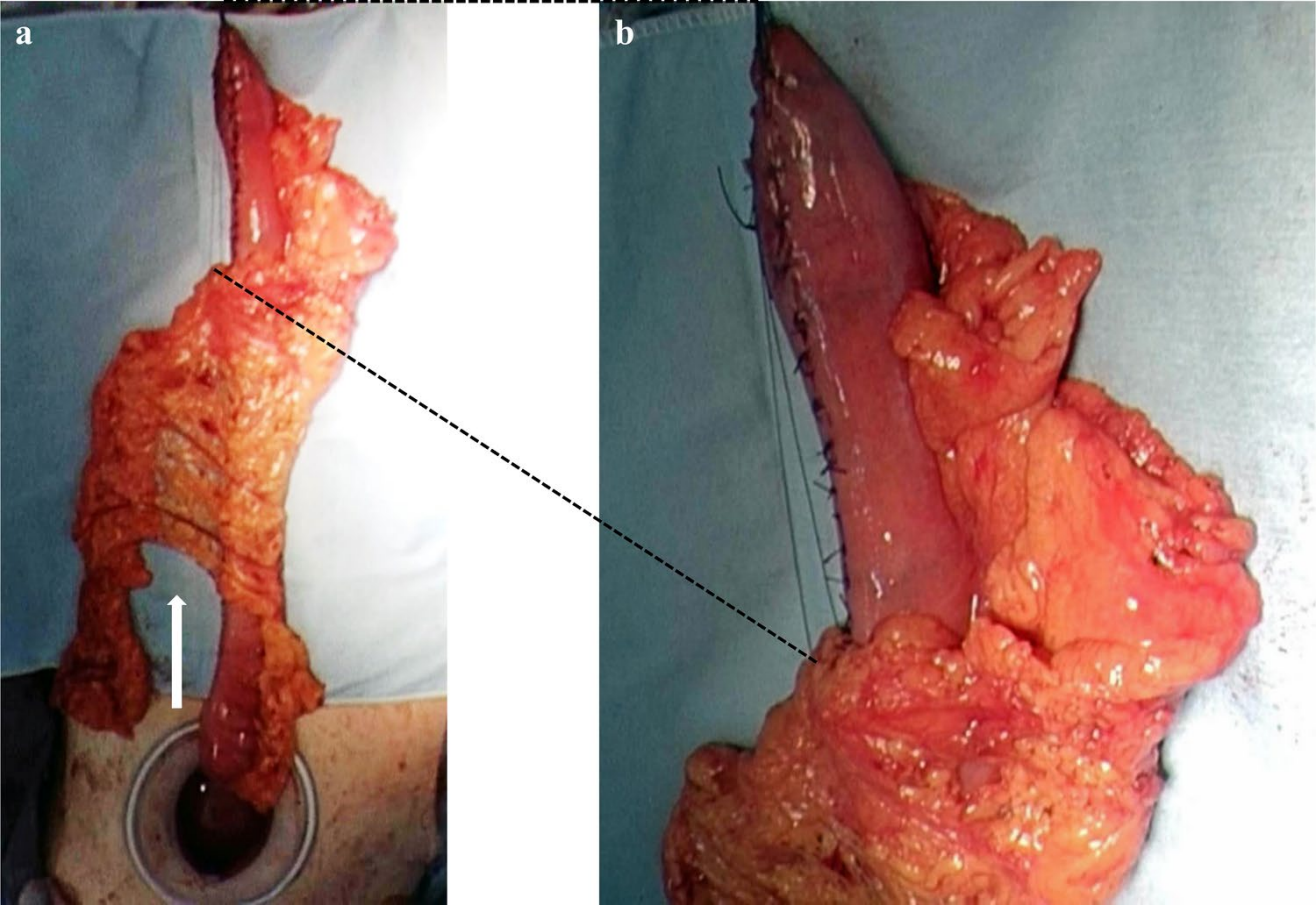
Omental flap and its fixation to the gastric tube for pulling up. **a** A sickle-shaped omental flap is made by cutting the rightmost and second epiploic vessels branching from the gastroepiploic vessels as shown by the white arrow (⇦). **b** Magnified photograph showing the upper third of the gastric tube. The tip of the omental flap is fixed with a thread to the tip of the gastric tube at least 15 cm below the top

Of 627 consecutive patients who underwent esophagectomy for esophageal cancer at our institution between January 2012 and March 2022, 325 patients had cT3 or more advanced thoracic esophageal cancer and underwent esophagectomy with reconstruction using a gastric conduit (Fig. 1). All patients had thoracic ESCC confirmed histologically by biopsy obtained during upper gastrointestinal endoscopy. Clinical staging was performed using computed tomography (CT) and ^1^⁸F-fluorodeoxyglucose positron emission tomography, based on the seventh edition of the UICC–AJCC staging system for esophageal and esophagogastric junction cancers [5]. All tumors were classified as cStage III or IV and treated according to the Japanese treatment guidelines for esophageal cancer [6]. This included preoperative chemotherapy for cT3 disease and induction or definitive chemoradiotherapy for cT4b disease.

Esophagectomy was performed when tumors were considered resectable or potentially resectable following induction therapy. In some cases, however, tumor infiltration into adjacent organs was identified intraoperatively. When infiltration was limited to connective tissue surrounding the airway or to the lung, combined resection was performed; otherwise, radical resection was abandoned. The application of dead space filling was determined based on intraoperative findings, particularly when the connective tissue of the membranous portion was deficient over a certain area and the respiratory muscle layer was exposed, or when the residual tumor ulcer bed remained exposed within the thoracic cavity. Ultimately, our new “waterfall” method was applied in 20 selected patients to prevent fatal postoperative complications. Surgical outcomes were retrospectively reviewed using medical records to assess the safety and usefulness of this technique.

This study was approved by the institutional review board of the Kindai University Faculty of Medicine (approval number R04-048).

### Preoperative or induction therapy

Standard triplet preoperative chemotherapy consisting of docetaxel (70 mg/m^2^) and cisplatin (70 mg/m^2^) administered on day 1, followed by continuous infusion of 5-fluorouracil (700 mg/m^2^/day) for 5 days (DCF regimen), was repeated every 3 weeks for up to three cycles depending on treatment response [7].

Elderly patients and those with impaired renal function received UDON triplet chemotherapy, a modified DCF regimen comprising docetaxel (35 mg/m^2^) on days 1 and 15, nedaplatin (90 mg/m^2^) on day 1, and continuous infusion of 5-fluorouracil (800 mg/m^2^/day) for 5 days. This regimen was repeated every 4 weeks for two cycles [8].

Induction and definitive chemoradiotherapy consisted of external beam radiotherapy with total doses of 50 Gy in 25 fractions over 5 weeks or 60 Gy in 30 fractions over 6 weeks, respectively, combined with concurrent chemotherapy using cisplatin (70 mg/m^2^) on days 1 and 29 and 5-fluorouracil (700 mg/m^2^/day) on days 1–4 and 29–32 [9].

### Evaluation of response to preoperative or induction therapy

The response of measurable lesions was assessed according to the Response Evaluation Criteria in Solid Tumors (RECIST) version 1.1 [10]. The response of the primary esophageal lesion was evaluated endoscopically in accordance with the 12th edition of the Japanese classification of esophageal cancer published by the Japan Esophageal Society [11]. Overall treatment response was categorized as complete response, partial response, stable disease, or progressive disease based on a comprehensive evaluation of target lesions, non-target lesions, the primary tumor, and the appearance of new lesions, in accordance with the Japanese classification system [11]. Tumor resectability was determined through multidisciplinary discussion.

### Surgical procedure

Esophagectomy was performed with the patient in the left decubitus position through a 10-cm mini-thoracotomy in the fourth intercostal space. Mediastinal dissection was carried out under magnified thoracoscopic visualization in all cases. When intraoperative findings suggested T4b disease, combined resection was performed according to the criteria described above; otherwise, radical resection was abandoned. After completion of the thoracic procedure, the patient was secured in a left semi-lateral decubitus position, allowing transition between lateral and supine operative fields through rotation of the operating table. In the supine position, the cervical procedure was performed via an open approach simultaneously with a hand-assisted laparoscopic abdominal approach. Gastric mobilization was performed while preserving as much omental tissue as possible.

A narrow gastric conduit approximately 4 cm in diameter was created, and a traction thread was secured to its tip for elevation to the neck. The rightmost and second right epiploic vessels were divided, and a notch was created in the greater omentum from the right side to form a partial sickle-shaped omental flap, thereby increasing its mobility (Fig. 1a). A separate thread was fixed to the tip of the omental flap and ligated to the traction thread of the gastric conduit at approximately 15 cm distal to the conduit tip (Fig. 1a and 1b). This ensured that the tip of the omental flap would be positioned in the center of the upper anterior mediastinum when the gastric conduit was elevated to the neck. The gastric conduit and omental flap were then placed into the plastic bag to prevent gastric conduit torsion and the omental flap was positioned to pass in front of the gastric conduit and lie on the right side, thereby facilitating its withdrawal into the thoracic cavity. Subsequently, the gastric conduit was lifted very carefully up to the neck along with the omental flap.

**Fig. 2:**
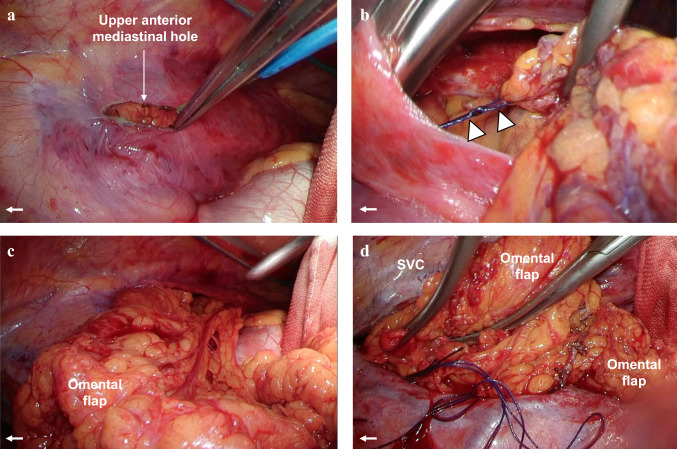
Incision of the upper anterior mediastinal pleura and pulling the omental flap down to the upper posterior mediastinum**. a** A small incision is made in the upper anterior mediastinal pleura. **b** The tip of the omental flap and its fixing thread (△) that were pulled up along with the gastric tube via the retrosternal route are visible in the center of the small incision. **c** After cutting the thread suspending the omental flap, the flap is pulled out via the small incision and placed in the upper portion of the posterior mediastinum. **d** The posterior mediastinal dead space is filled using the omental flap after esophagectomy by placing the flap. *SVC* superior vena cava, White arrows (⇦) in all images: the cranial side

**Fig. 3:**
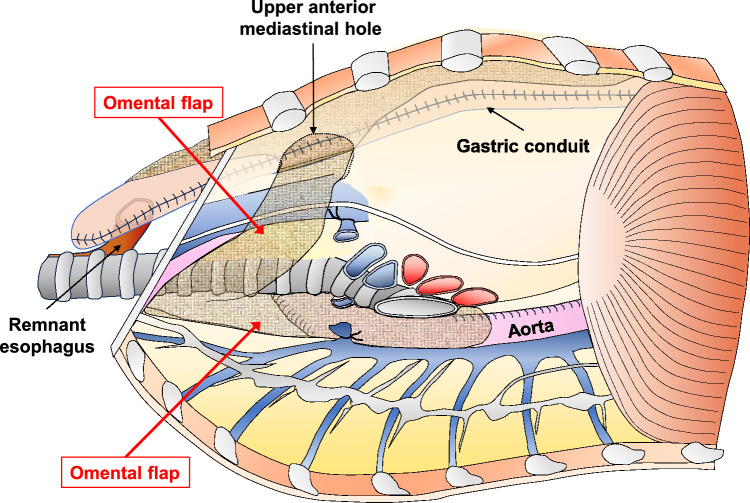
Illustration after completion of the “waterfall” technique. The greater omental flap is pulled up along with the gastric conduit via the retrosternal route, pulled down from a small incision in the upper anterior mediastinal pleura into the posterior mediastinum, and then used to fill into the dead space after esophagectomy. This figure was created by modifying and adapting an illustration used in our paper accepted for publication in the Journal of Thoracic and Cardiovascular Surgery [25]

The operating table was again rotated to place the patient in the left lateral decubitus position for a second thoracic procedure. A small incision was made in the mediastinal pleura of the upper anterior mediastinum, where the elevated gastric conduit and omental flap were visualized (Fig. 2a). After cutting the ligating thread at the tip of the gastric conduit (Fig. 2b), the omental flap was gently pulled into the right thoracic cavity (Fig. 2c). The omentum pulled down from the anterior mediastinum was then used to fill the posterior mediastinal dead space created by esophagectomy (Figs. 2d and 3). The thoracic cavity was subsequently closed.

Finally, after returning the patient to the supine position, cervical esophagogastric anastomosis was performed using a circular stapler (23–25 mm in diameter).

### Short-term postoperative complications

Short-term postoperative complications were evaluated and graded according to the Common Terminology Criteria for Adverse Events (CTCAE) version 5.0 [12]. Delayed gastric emptying was assessed using the symptom grading tool recommended by an international expert consensus for patients undergoing esophagectomy for cancer [13]. Delayed gastric emptying was diagnosed when at least two of the following five symptom categories were rated as “quite a bit” or “very much”: early satiety or fullness, vomiting, nausea, regurgitation, or inability to achieve adequate caloric intake orally.

## Results

### Patient characteristics

Patient characteristics and surgical outcomes for the 20 patients (15 men and 5 women) who underwent the waterfall method are summarized in Table 1. Patient age ranged from 46 to 76 years, and all tumors were histologically confirmed as squamous cell carcinoma. Initial clinical tumor stage was cT3 in 4 patients and cT4b with invasion of the aorta or airway in 16 patients.

**Table 1:** Patient characteristics and surgical outcomes

**Characteristics**		**Number**
No. of patients		20
Sex	Male	15
	Female	5
Age (years)	Median (range)	65.3 (46 ~ 76)
Tumor location	Ut	11
	Mt	7
	Lt	2
Histology	SCC	20
cT	cT3	4
	cT4b(Aorta)	5
	cT4b(airway)	11
cStage	IIIA	1
	IIIB	1
	IIIC	12
	IV	6
Preoperative / Induction therapy	CTx	10
	CRT	4
	CTx → CRT	2
	CRT → CTx	4
Response	CR	1 (recurrence)
	PR	14
	SD	4
	PD	1
Operative time	median (range)	792 min. (640 ~ 990)
Blood loss	Median (range)	626 ml (280 ~ 1614)
Residual tumor	R0	12
	R1	0
	R2	8
Cause of omentoplasty	Combined resection	12
	R2 resection	8
Short-term surgical outcomes	Mediastinitis	0
	Fistula / Airway necrosis	0
	Bleeding	0
	Pneumothorax	3
	Pneumonia	3
	Anastomotic leakage	2
Long-term outcomes	Delayed gastric emptying	0
Postoperative in-hospital stay	median (range)	47 days (18 ~ 298)

*CTx* chemotherapy, *CRT* chemoradiation, *min* minutes, *CR* complete response, *PR* partial response, *PD* progressive disease, *R0* no residual tumor, *R1* microscopic residual tumor, *R2* macroscopic residual tumor, *SCC* squamous cell carcinoma, *SD* stable disease, *Ut/Mt/Lt* upper/middle/lower thoracic esophagus

Preoperative or induction therapy consisted of chemotherapy alone in 10 patients, chemoradiotherapy in 4 patients, and a combination of both in 6 patients, resulting in an overall response rate of 75%. The median operative time and blood loss were 792 min and 626 ml, respectively. Curative (R0) resection was achieved in 12 patients, including combined resection of peritracheobronchial connective tissue in 11 patients and right lower lobectomy in one patient (Supplementary Table 1). The remaining 8 patients underwent R2 resection. The median postoperative in-hospital stay was 47 days.

### Indications for omental filling of the dead space

As summarized in Table 1 and Supplemental Table 1, indications for omental filling were reinforcement of the vulnerable airway membranous portion following resection of peritracheobronchial connective tissue (*n* = 11), protection of the bronchial stump after combined resection of the right lower lung (*n* = 1), and prevention of progression and spread of infection originating from residual tumor tissue after R2 resection (*n* = 8).

### Short-term surgical outcomes

No serious postoperative complications anticipated intraoperatively—such as tracheobronchial necrosis or stenosis, airway–mediastinal fistula, massive bleeding, infectious mediastinitis, or mediastinal abscess—were observed (Tables 1 and Supplementary Table 1). No complications related to omentoplasty, including omental necrosis, infection, or bleeding, occurred.

Anastomotic leakage (CTCAE grade 2) occurred in two patients, pneumothorax (grade 2) in three, aspiration pneumonia (grade 2) in one, and pneumonia (grade 4) in two patients. Importantly, no adverse events were attributable to the waterfall method itself, and there were no surgery-related deaths.

### Long-term outcomes

**Fig. 4:**
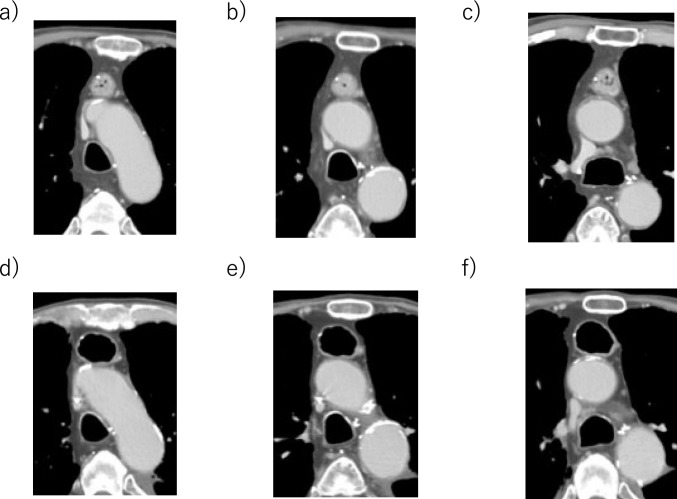
Axial thoracic computed tomography images for case 20 in Table 1. **a**–**c** Images obtained 3 months after surgery. **d**–**f** Images obtained 45 months after surgery

In the patient with the longest follow-up (45 months), mild atrophy of the greater omentum was observed, while adequate airway coverage was maintained on computed tomography (Fig. 4). No cases of dilation, flexion, or deviation of the gastric conduit into the thoracic cavity were observed. Consequently, no patients developed delayed gastric emptying with severe symptoms according to the international consensus diagnostic criteria, although some patients reported mild sensations of satiety or fullness. No cases of impaired gastric conduit transit related to conduit configuration or local recurrence were observed, except for general deterioration associated with disease progression in R2 cases.

## Discussion

We developed a novel surgical technique, termed the “waterfall method”, to fill the posterior mediastinal dead space created after esophagectomy by transposing the greater omentum via the retrosternal route rather than the conventional posterior mediastinal route. In this procedure, the omental flap is elevated to the neck together with the gastric conduit and subsequently pulled down from the anterior mediastinum to fill the posterior mediastinal cavity. We refer to this technique as the “waterfall method” because the greater omentum naturally drapes downward along the gastric conduit, covering the posterior mediastinal space in a manner resembling a waterfall. This technique was applied in 20 selected patients who required combined resection of infiltrated tissues or who underwent R2 resection. No serious postoperative complications—such as tracheobronchial necrosis, airway collapse, or exacerbation of mediastinal infection—were observed, suggesting that the greater omentum may function effectively as a reinforcing and space-filling tissue.

After esophagectomy, resection of the surrounding peritracheobronchial tissues may result in loss of structural support for the membranous portion of the airway, leaving it exposed over the post-esophagectomy cavity. Because this cavity can change in volume with respiration, the unsupported membranous portion may be subjected to dynamic deformation driven by airway pressure fluctuations. In the absence of normal connective tissue restraint, the thin residual layer composed of mucosa and smooth muscle may bulge inward during inspiration and distend outward during expiration, particularly during forceful respiration or coughing. Such repetitive deformation could potentially compromise microvascular perfusion of the membranous portion, thereby increasing the risk of ischemia and subsequent airway–mediastinal fistula formation. The tracheal mucosa is known to be vulnerable to ischemic injury caused by mechanical compression, as demonstrated in studies of cuff-related tracheal injury during prolonged intubation. In such settings, mucosal ischemia may progress to tracheoesophageal fistula formation, as previously reported [14]. In cases of R2 resection, if a contaminated ulcer bed of residual tumor remains within the mediastinum, accumulation of postoperative exudate promotes bacterial proliferation, placing the patient at high risk for severe mediastinitis. In both scenarios, it is essential to obliterate the dead space after esophagectomy with well-vascularized tissue to reinforce the airway and eliminate a potential nidus for infection.

An ideal tissue for filling the dead space should have abundant blood supply, resistance to infection, and sufficient volume to pack the cavity. Although muscles such as the latissimus dorsi or pectoralis major have been used for this purpose, the greater omentum offers several advantages, including rich vascularity, immunologic activity due to abundant milky spots [15], angiogenic potential [16], and absorptive capacity for excess fluid [17]. These properties have led to its use in the treatment of empyema and mediastinitis, reinforcement of bronchial stumps after pulmonary resection [18, 19], and prevention of anastomotic leakage after esophagectomy [20, 21]. In addition, the greater omentum produces vascular endothelial growth factor [22], facilitating revascularization and tissue healing [23, 24]. Given these characteristics, the greater omentum is particularly well suited for reinforcement and infection control in the posterior mediastinum.

Although the dead space after esophagectomy is located in the posterior mediastinum, the high mobility of the omental flap allows transposition from the anterior mediastinum even when the gastric conduit is reconstructed via the retrosternal route. Our waterfall method successfully achieved this in all patients without serious complications such as airway fistula, tracheobronchial stenosis due to compromised blood flow, or deep mediastinitis. A major advantage of this technique over the conventional posterior mediastinal approach is that the gastric conduit is positioned centrally within the anterior mediastinum and outside the thoracic cavity. This configuration protects the conduit from intrathoracic negative pressure, preventing dilation, displacement, and regurgitation, thereby ensuring favorable postoperative transit. In addition, the conduit remains uninvolved in the event of local recurrence or regrowth of residual tumor, and postoperative radiotherapy can be administered without concern for conduit positioning. Because of its technical simplicity, this procedure is well suited for minimally invasive esophagectomy, including thoracoscopic or robotic approaches.

Good candidates for this technique include patients who are at high risk of developing gastro-airway fistula, such as those who have undergone resection of the connective tissue surrounding the membranous portion of the airway, those with a bronchial stump after simultaneous lung lobectomy, or those with contaminated residual tumor ulcer beds that may promote mediastinal infection and subsequent fistula formation. In the present series, no cases of airway–mediastinal fistula were observed, although the relatively small number of patients precludes definitive conclusions.

Several technical points are essential for successful application of this method. First, a notch should be created on the right side of the greater omentum by dividing the rightmost and second epiploic vessels to form a sickle-shaped flap (Fig. 1a), thereby maximizing reach into the thoracic cavity (Figs. 2d and 3). Second, the tip of the omental flap should be fixed with a thread at least 15 cm distal to the tip of the gastric conduit to ensure correct positioning in the anterior mediastinum during elevation of the gastric conduit (Fig. 1b). If the fixation point is too proximal, the omental flap may become impacted in the narrow thoracic inlet together with the gastric conduit, resulting in considerable difficulty in pulling the omental flap downward. This represents an important technical point to avoid during the procedure. Third, the omental flap should be placed to the right, passing in front of the gastric conduit, to facilitate smooth transposition. However, because the greater omentum is originally attached to the greater curvature of the stomach on the left side, a theoretical concern exists regarding the potential risk of gastric conduit torsion. In practice, the gastric conduit is stabilized within the retrosternal space, and only the omental flap located on the right side of the conduit is gently drawn down into the right thoracic cavity. Because excessive traction is not required, this procedure is considered unlikely to cause gastric conduit torsion, and no such complication was observed in the present series. Fourth, creation of the retrosternal tunnel and the pleural incision in the anterior mediastinum should be performed carefully and kept as small as possible to minimize the influence of intrathoracic negative pressure (Fig. 2a). Finally, even in lean patients, the volume of the greater omentum is usually sufficient to fill the posterior mediastinal dead space after esophagectomy. However, if the omental volume is insufficient, alternative approaches such as the use of a pedicled muscle flap may be necessary.

This study has several limitations, including its retrospective, single-center design, small sample size, and lack of objective quantitative assessment of delayed gastric emptying. In particular, objective functional or morphological assessment of the gastric conduit would be desirable in future studies. Moreover, it is ethically difficult to determine whether this technique definitively prevents serious postoperative complications compared with no dead space filling or the conventional method using posterior mediastinal reconstruction. This study is a retrospective single-arm experience, and the absence of a control group may introduce potential bias in evaluating the true efficacy of the technique. Furthermore, the target population is relatively rare, and severe complications such as tracheal membranous rupture are often life-threatening, making controlled comparative studies challenging in clinical practice. In recent years, retrosternal reconstruction has been increasingly adopted because of its potential advantages, including a lower risk of gastro-tracheal fistula, reduced reflux, and easier creation of a gastrostomy. Therefore, rather than switching to posterior mediastinal reconstruction only when omental filling is required, it may be advantageous to have a technique that allows omental filling even during standard retrosternal reconstruction.

In conclusion, the waterfall method is a simple, feasible, and potentially safe technique that allows effective filling of the posterior mediastinal dead space while preserving optimal positioning and function of the gastric conduit. This approach may represent a valuable option for surgeons managing complex cases of esophageal cancer requiring combined resection or non-curative surgery. Further clinical experience will help to better define the indications and clinical utility of this technique.

## Supplementary Information

Below is the link to the electronic supplementary material.

Supplementary file1 (PDF 37 KB) Supplemental Fig. 1 CONSORT flow diagram showing the patient selection process

Supplementary file2 (PDF 121 KB)
