# Supplementary figures and images for: Retrosternal gastric reconstruction after esophagectomy using the “waterfall” method for posterior mediastinal dead space filling
Source: Esophagus. 2026 Apr 8;23(3):562–70. doi: 10.1007/s10388-026-01204-4 (PMC13319266; doi:10.1007/s10388-026-01204-4)

## Supplemental Fig.1

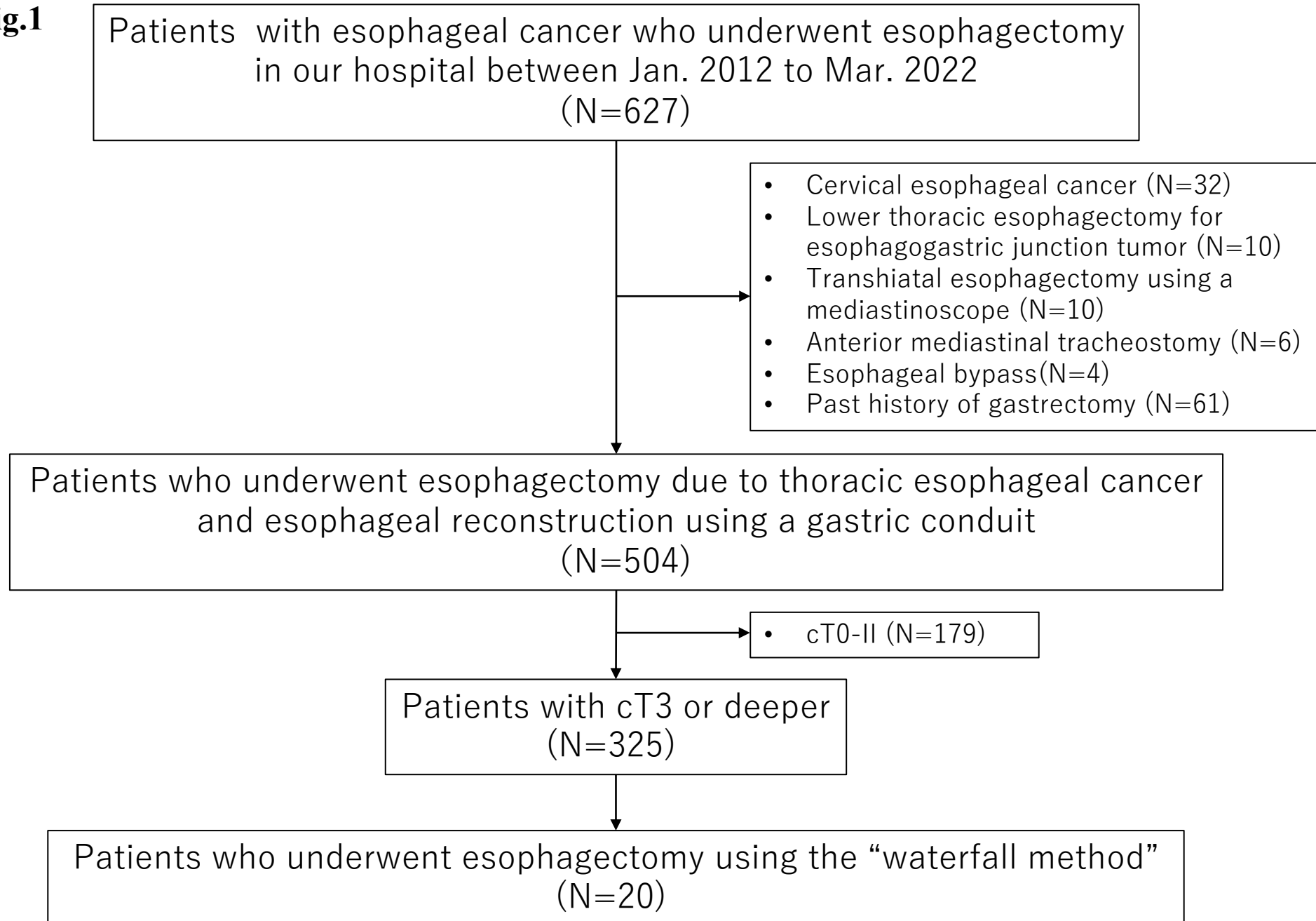

Supplement: Supplementary file 1 — Supplementary file1 (PDF 37 KB) Supplemental Fig. 1 CONSORT flow diagram showing the patient selection process [file 10388_2026_1204_MOESM1_ESM.pdf]
