# Supplementary material for: Retrosternal gastric reconstruction after esophagectomy using the “waterfall” method for posterior mediastinal dead space filling
Source: Esophagus. 2026 Apr 8;23(3):562–70. doi: 10.1007/s10388-026-01204-4 (PMC13319266; doi:10.1007/s10388-026-01204-4)
Supplement: Supplementary file 2 — Supplementary file2 (PDF 121 KB) [file 10388_2026_1204_MOESM2_ESM.pdf]

Supplemental Table 1. Clinical course and surgical outcomes of each case

**Case**	**Year<br>Age<br>Sex**	**Tumor location**	**cStage/<br>cTNM**	**Preoperative or induction therapy**	**Res-<br>ponse**	**Resi-<br>dual<br>tumor**	**Reason of omentoplasty**	**Postoperative complication**					
								mediast<br>initis	Fistula/<br>Airway<br>necrosis	Bleed<br>ing	AL	Respiratory	DGE
**1**	2012<br>67<br>M	MtUt	IIIC/<br>T4b(lt-mBr)<br>N1M0	50Cy iCRT	PR	R2<br>(lt-mBr)	R2	(-)	(-)/(-)	(-)	(-)	(-)	(-)
**2**	2013<br>66<br>M	UtMt	IIIC/<br>T4b(Ao)<br>N1M0	50Gy iCRT<br>→TEVER	PR	R2	R2	(-)	(-)/(-)	(-)	(-)	Pneumo-<br>thorax<br>G2	(-)
**3**	2013<br>59<br>M	Ut	IIIC/<br>T4b(Tr)<br>N1M0	(FP×①)※<br>→50Gy iCRT	PR	R2<br>(Tr)	R2	(-)	(-)/(-)	(-)	(-)	Pneumo-<br>thorax<br>G2	(-)
**4**	2014<br>66<br>M	Ut	IIIC/<br>T4b(Tr)<br>N0M0	(60Gy dCRT→FP×<br>⑤→CR→Rec.)※	CR	R0	CTR of the membranous trachea	(-)	(-)/(-)	(-)	(-)	Aspiration pneumonia<br>G2	(-)
**5**	2014<br>66<br>M	UtMt	IV/<br>T4b(Ao)<br>N0M1LYM	50Gy iCRT	PR	R2<br>(Ao)	R2	(-)	(-)/(-)	(-)	(-)	(-)	(-)
**6**	2014<br>47<br>M	UtMt	IIIC/<br>T4b(Ao)<br>N1M0	60Gy dCRT<br>→FP×①	PR	R0	CTR of the membranous portion of the lt-mBr	(-)	(-)/(-)	(-)	(-)	(-)	(-)
**7**	2014<br>68<br>F	Lt	IV/<br>T4b(rt-lung)<br>N2M1LYM	(40Gy CRT→PE)※<br>→DCF×①	SD	R0	Combined resection of the right lower lung	(-)	(-)/(-)	(-)	(-)	(-)	(-)
**8**	2015<br>71<br>M	Mt	IV/<br>T3<br>N2M1LYM	DCF×③	PR	R0	CTR of the membranous trachea	(-)	(-)/(-)	(-)	(+)<br>G2	Pneumonia<br>G4	(-)
**9**	2016<br>62<br>M	UtMt	IIIC/<br>T4b(Ao)<br>N0M0	DCF×②	SD	R2<br>(Ao~lt-<br>mBr)	R2	(-)	(-)/(-)	(-)	(-)	(-)	(-)
**10**	2016<br>76<br>F	UtMt	IIIC/<br>T4b(Ao)<br>N2M0	(DCF×③)※	PR	R2<br>(Ao)	R2	(-)	(-)/(-)	(-)	(-)	(-)	(-)

**11**	2016<br>70<br>M	Ut	IV/<br>T4b(Tr)<br>N2M1LYM	(60Gy dCRT→FP×②)※→DCF×①	PR	R0	CTR of the membranous trachea	(-)	(-)/(-)	(-)	(-)	RLN palsy/<br>pneumonia<br>G4	(-)
**12**	2016<br>63<br>M	Mt	IIIC/<br>T4b(Tr)<br>N0M0	UDON×②	PR	R0	CTR of the membranous trachea	(-)	(-)/(-)	(-)	(-)	(-)	(-)
**13**	2017<br>68<br>F	Mt	IV/<br>T4b(Carina)<br>N1M1LYM	60Gy dCRT	SD	R2<br>(Tr)	R2	(-)	(-)/(-)	(-)	(-)	(-)	(-)
**14**	2017<br>73<br>M	LtMt	IIIA/<br>T3<br>N1M0	(FP×①<br>→DCF×①)※	PD	R2<br>(Ao~lt-mBr)	R2	(-)	(-)/(-)	(-)	(-)	(-)	(-)
**15**	2017<br>67<br>M	Ut	IIIC/<br>T4b(Tr)<br>N2M0	DCF×②	PR	R0	CTR of the membranous trachea	(-)	(-)/(-)	(-)	(+)<br>G2	(-)	(-)
**16**	2018<br>74<br>F	Mt	IIIB/<br>T3<br>N2M0	DCF×②	SD	R0	CTR of membranous portion of the carina and the rt-mBr	(-)	(-)/(-)	(-)	(-)	(-)	(-)
**17**	2018<br>75<br>M	Mt	IV/<br>T3<br>N2M1LYM	UDON×②→PR→<br>Ope reject→regrowth	PR	R0	CTR of membranous portion of the lt-mBr	(-)	(-)/(-)	(-)	(-)	Pneumo-thorax<br>G2	(-)
**18**	2021<br>46<br>F	Ut	IIIC/<br>T4b(Tr)<br>N1M0	DCF×①<br>→50Gy iCRT	PR	R0	CTR of the membranous trachea	(-)	(-)/(-)	(-)	(-)	(-)	(-)
**19**	2021<br>55<br>M	Mt	IIIC/<br>T3<br>N3M0	(DCF×③<br>→PR→regrowth)※	PR	R0	CTR of membranous portion of the lt-mBr	(-)	(-)/(-)	(-)	(-)	(-)	(-)
**20**	2022<br>67<br>M	UtCe	IIIC/<br>T4b(Tr)<br>N1M0	DCF×②	PR	R0	CTR of the membranous trachea	(-)	(-)/(-)	(-)	(-)	(-)	(-)

*Conducted at another hospital. AL: anastomotic leakage, Ao: aorta, CTR: connective tissue resection, CR: complete response, DCF: docetaxel+cisplatin+5-fluorouracil, dCRT: definitive chemoradiotherapy, DGE: delayed gastric emptying, F: female, FP: 5-fluorouracil+cisplatin, G: Grade (CTCA ver.5.0), iCRT: induction chemoradiotherapy, LND: lymph node dissection, lt-mBr: left main bronchus, M: male, N.A.: not applicable, Ope: operation, PD: progressive disease, PE: pulmonary embolism, PR: partial response, R0: no residual tumor, R2: macroscopic residual tumor, Rec: recurrence, RLN: recurrent laryngeal nerve, rt-lung: right lung, SD: stable disease, TEVAR: thoracic endovascular aortic repair, Tr: trachea, UDON: 5-fluorouracil+docetaxel+nedaplatin, Ut/Mt/Lt: upper/middle/lower thoracic esophagus
